# Origin of clay minerals in Early Eocene volcanic paleosols on King George Island, Maritime Antarctica

**DOI:** 10.1038/s41598-017-06617-x

**Published:** 2017-07-25

**Authors:** Diogo Noses Spinola, Teresa Pi-Puig, Elizabeth Solleiro-Rebolledo, Markus Egli, Masafumi Sudo, Sergey Sedov, Peter Kühn

**Affiliations:** 1 Department of Geosciences, Research Area Geography, University of Tübingen, Tübingen, Germany; 2 Instituto de Geología, Universidad Nacional Autónoma de México, México, D.F Mexico; 3 Department of Geography, University of Zurich, Zurich, Switzerland; 4 Institute of Earth and Environmental Science, University of Potsdam, Potsdam, Germany

## Abstract

The paleoclimate during the Early Eocene in Maritime Antarctica is characterized by cool conditions without a pronounced dry season. Soils formed on volcanic material under such climate conditions in modern analogue environments are usually Andosols rich in nanocrystalline minerals without pedogenic smectite. The paleosols formed on volcanic material on King Georges Island are covered by basalts, dated by 6 new ^40^Ar/^39^Ar datings to 51–48 Ma, and are rich in smectite. A pedogenic origin of the smectites would suggest a semi-arid rather than a wet non-seasonal humid paleoclimate. To investigate the origin of the smectites in these paleosols we used X-ray diffraction and microscopic techniques. Minor mineralogical changes between the volcanic parent material and the paleosols and a homogenous distribution of smectites throughout the paleosol horizons indicate that these smectites were mainly inherited from the pyroclastic parent material, which was altered prior to surficial weathering. Nevertheless, the mineralogical properties, such as degree of crystallinity and octahedral site occupancy, of these smectites were modified during the ancient soil formation. Our findings highlight that trioctahedral smectites were a product of deuteric alteration of pyroclastic rocks and were progressively transformed to dioctahedral smectites during weathering in a soil environment on King George Island.

## Introduction

The paleoclimate during the Early Eocene in Maritime Antarctica is characterized by cool conditions without a pronounced dry season. Studies based on plant fossil assemblages suggest a paleoenvironment similar to the Valdivian rainforest in southern Chile^1–3^. The Valdivian rainforest is considered as a suitable modern analogue because of similar plant assemblages and because of volcanic substrates^1^.

Andosols are the predominant type of volcanic soils formed under a cool temperate environment, like the Valdivian rainforest. Nanocrystalline minerals (i.e. allophane and ferryhydrite) and imogolite are the predominant secondary minerals in these soils^4–7^. In environments having a pronounced dry-season, the occurrence of halloysite and smectite becomes more common^8, 9^. So far the origin of smectites within this material is not clarified and mainly three different origins are proposed: (i) many authors suggest an inheritance origin, when the volcanic substrate was subject to a hydrothermal or deuteric alteration (a low-temperature magmatic alteration related to the solidification of a melt) leading to a formation of smectites prior to a subsequent surficial weathering^10–15^. (ii) Smectites are of pedogenic origin as a product of *in-situ* weathering of primary minerals^16–20^. (iii) Aeolian input of smectites may have played a major role^21, 22^.

The importance of clay mineralogy for paleoenvironmental interpretation is well recognized^23, 24^. Nonetheless, clay minerals are only suitable for a paleoenvironmental interpretation when their origin in paleosols is confirmed as being pedogenic^25^. For this reason, before interpreting clay minerals as weathering products of the tephra, a potential hydrothermal or deuteric and/or aeolian origin must be excluded.

King George Island (KGI), Maritime Antarctica, is one of the few sites in Antarctica where Eocene paleosols were recognized. In a first attempt for a paleoenvironmental reconstruction based on its clay mineralogy, Birkenmajer and Łydka (1990)^26^ attributed the presence of smectite and kaolinite in paleosols as a record of a warm-wet paleoclimate. However, no further studies were made to understand the origin of these clays. In a recent publication, smectite was identified in Eocene paleosols on KGI^27^. A predominance of smectite, however, should not exist, because these paleosols correlate with fossil plants layers that indicate a cool paleoenvironment. Thus, to avoid misinterpretations of the Eocene paleoclimate using the clay mineralogy of these paleosols, the origin of these clay minerals must be understood.

The objective of this study was, therefore, to investigate – by using X-ray diffraction, microscopic and sub-microscopic techniques (SEM-EDS) - the origin of smectites in Eocene paleosols on KGI. The occurrence of smectite in these paleosols have a potential to give additional paleoenvironmental information, e.g. pedogenic smectites would suggest a dryer climate for the Early Eocene which would be in contrast to the wet non-seasonal paleoclimate model^1–3, 28^.

## Results

### Field – General characteristics

**Figure 1:**
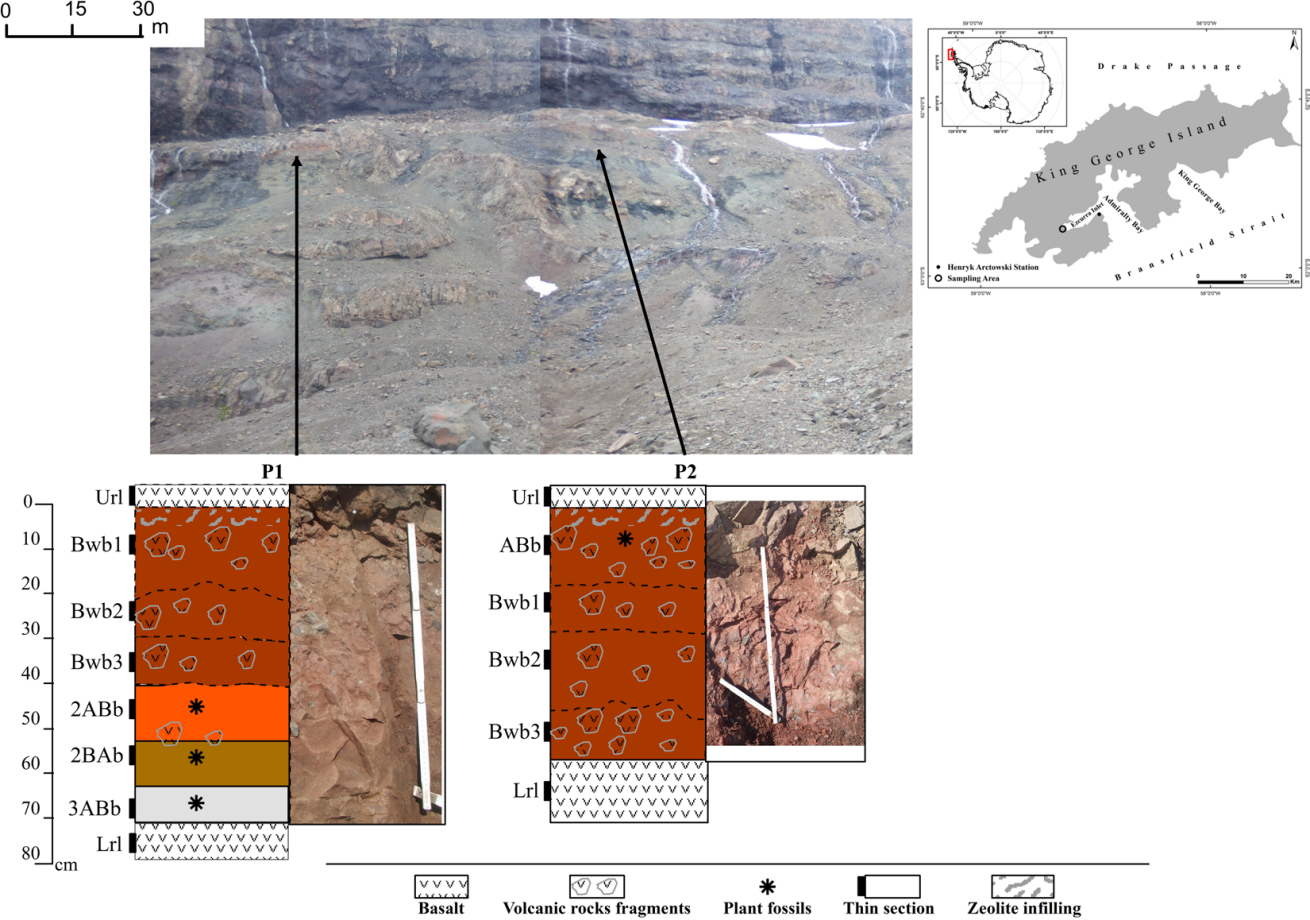
Overview of the sampling site at King George Island, location of the paleosols at the Cytadela outcrop and their schematic profile description (modified from Spinola *et al*.^27^).

The two investigated profiles are morphologically similar (Supplementary Table T1). Both are relatively thick paleosols profiles (P1: > 73 cm, P2: > 56 cm) occurring between basalt flows (Fig. 1). Whereas the contact with the covering basalt flow is clear, the contact with the saprolite and the underlying basalt was not accessible. Both profiles are reddish-brown with gradual colour changes with depth. They are characterized by a predominantly strong blocky (more angular) and platy structure and a variable size of aggregates with depth. Most of the horizons have a sandy loam texture (Table 1). The 2BAb and 3ABb horizons of P1 and ABb of P2 have a slightly finer texture with a sandy clay loam.

**Table 1:** Grain-size distribution, textural classes and smectite properties and proportion.

Profile	Horizon	Depth	Particle size [%]			Textural class	Octahedral occupancy of smectites [%]		Smectite in the parent material [%]
		[cm]	Clay (<2 µ)	Silt (2 µ-63 µ)	Sand (63 µ−2000 µ)		Dioctahedral	Trioctahedral	
P1	Bwb1	0–23	15	18	67	Sandy Loam	92	8	
	Bwb2	23–34	15	19	66	Sandy Loam	97	3	
	Bwb3	34–45	14	19	67	Sandy Loam	92	8	
	Parent material	from Bwb3 horizon					55	45	28.4
	2Abb	45–60	17	21	62	Sandy loam	81	19	
	2Bab	60–73	20	22	58	Sandy clay loam	91	9	
	Parent material	from 2ABb horizon					68	32	33.6
	3Abb	73+	20	15	65	Sandy clay loam	99	1	
	Parent material	from 3ABb horizon					72	28	41.8
P2	Abb	0–20	19	17	64	Sandy clay loam	98	2	
	Bwb1	20–30	15	19	66	Sandy loam	98	2	
	Bwb2	30–53	15	18	67	Sandy loam	99	1	
	Bwb3	53+	15	13	72	Sandy loam	97	3	
	Parent material	from Bwb3 horizon					75	25	29.7

Based on the colour, P1 is the more heterogeneous profile. The colours vary from red to grey, between structure type and size, and the rock fragments in the horizons. It is also thicker than P2 and has two lithic discontinuities (LD). The first LD is between the Bwb3 and 2ABb horizons due to an abrupt lack in rock fragments in the 2ABb horizon. The second LD is between the 2BAb and 3ABb horizons, indicated by the appearance of dark fossil plant leaves and a greyish colour (hue = 5Y) being a marker of a buried A horizon. The horizon boundaries in P2 are gradual and diffuse. The horizons can be distinguished mostly by differences in the structure type that varies between subangular/angular blocky and platy. P2 has a predominantly reddish-brown (hue-2.5YR) colour.

### ^40^Ar/^39^Ar dating

**Figure 2:**
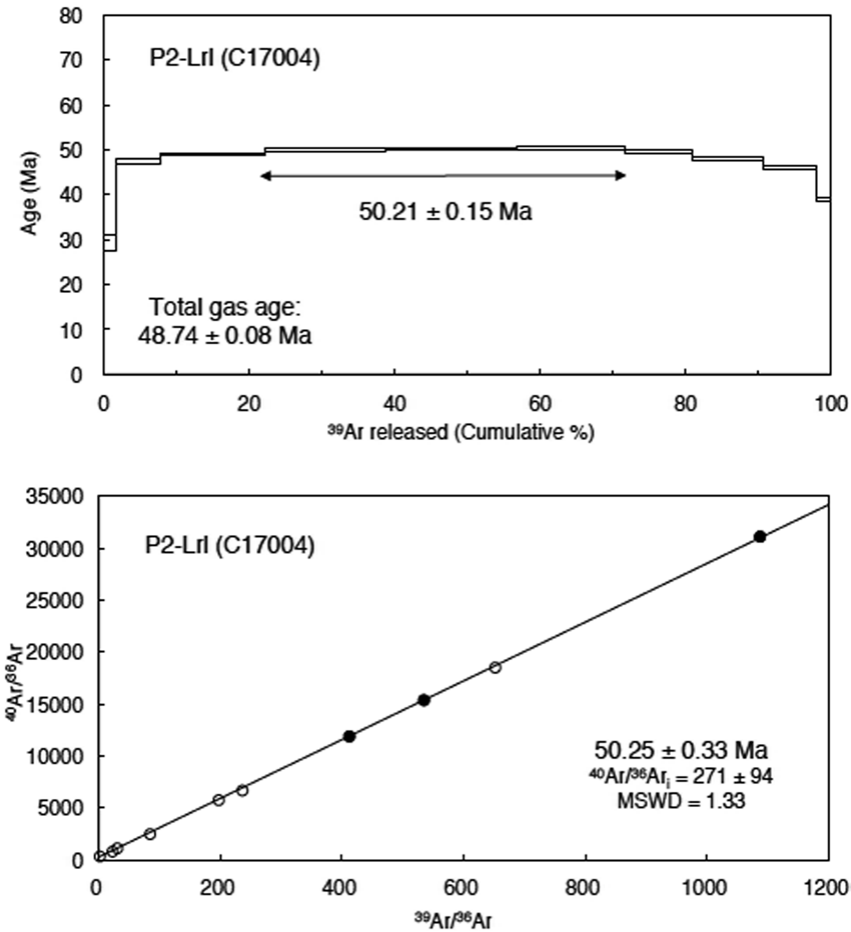
^40^Ar/^39^Ar age spectrum and normal isochron plot obtained by the stepwise heating analysis of groundmass sample, P2-Lrl. C17004: Laboratory-ID. The error in the age spectrum shows 1 sigma error. Closed circles in the isochron plot are derived from plateau steps while open circles are not. Plateau age and isochron age from the plateau steps correspond well.

The ^40^Ar/^39^Ar age of the lava flows confirms a late Early Eocene age (Fig. 2, Table 2, Supplementary Fig. F1 and Supplementary Table T2). The ages of the basalts covering the profiles should be identical because they belong to the same lava flow (Fig. 1), within 60 m horizontally from P1 to P2 and also 90 m from P2 to P3 (not studied in the present manuscript). The ages from upper (Url) and lower (Lrl) basaltic lava flows at P1 (P1-Url and P1-Lrl) are 50.47 ± 0.08 (error: 1 sigma, Lab ID: C16047) and 48.57 ± 0.29 Ma (C17003). The ages are similar, although the upper flow is slightly older than the lower one beyond 2 sigma error. The ages from the upper and lower basaltic lava flows at P2 (P2-Url and P2-Lrl) are 42.66 ± 0.20 (C16048) and 50.21 ± 0.15 Ma (C17004). Thus, the age only from P2-Url is much younger than the other three ages from 50.5 to 48.5 Ma. Especially the result of sample P2-Lrl is excellent result because the plateau age and isochron ages from the plateau steps correspond very well (Fig. 2 and Table 2). Therefore, the age 50.21 Ma of P2-Lrl will be used as the best inferior age for the basaltic units. The younger age of 42.66 Ma from P2-Url could be explained by the larger loss of radiogenic ^40^Ar from the sample due to alteration, which is consistent with the petrographic observation, and the Ar loss curve in the lower temperature steps in the age spectrum (Supplementary Fig. F1). For the ages of P1-Url and P3-Url is the most probable age for the upper basaltic unit 50.5 to 48 Ma. Therefore, the new ^40^Ar/^39^Ar datings propose an age for the basalt flows ranging between 51 Ma and 48 Ma, thus, dating to the late Early Eocene.

**Table 2:** ^40^Ar/^39^Ar analytical result of basaltic lava flow, P2-Lrl.

Laser output	^40^Ar/^39^Ar	^37^Ar/^39^Ar	^36^Ar/^39^Ar	K/Ca	^40^Ar*	^39^Ar_K_	^40^Ar*/^39^Ar_K_	Age (±1 s)
			(×10^−3^)		(%)	fraction (%)		(Ma)
***Sample ID:P2-Lrl***		*Laboratory ID: C17004*		*Neutron irradiation ID: PO-5*				
J = 0.000999								
1.4%	190.81 ± 1.48	0.535 ± 0.011	590.75 ± 5.02	1.10	8.53	1.58	16.29 ± 0.99	29.13 ± 1.76
1.6%	36.36 ± 0.35	0.520 ± 0.005	32.95 ± 0.33	1.13	73.34	6.28	26.68 ± 0.31	47.45 ± 0.57
1.8%	29.11 ± 0.12	0.515 ± 0.004	5.21 ± 0.05	1.14	94.85	14.21	27.62 ± 0.11	49.11 ± 0.28
2.0%	28.86 ± 0.14	0.562 ± 0.004	2.58 ± 0.04	1.05	97.51	16.67	28.15 ± 0.14	50.03 ± 0.31
2.2%	28.74 ± 0.09	0.667 ± 0.004	2.05 ± 0.03	0.88	98.08	18.11	28.20 ± 0.09	50.13 ± 0.25
2.4%	28.61 ± 0.08	0.818 ± 0.004	1.14 ± 0.04	0.72	99.05	14.72	28.35 ± 0.08	50.38 ± 0.24
2.6%	28.38 ± 0.12	0.895 ± 0.008	1.78 ± 0.06	0.66	98.40	9.40	27.94 ± 0.12	49.67 ± 0.29
2.9%	28.23 ± 0.15	0.967±0.007	4.50 ± 0.07	0.61	95.57	9.57	27.00 ± 0.15	48.02 ± 0.32
3.3%	29.34 ± 0.15	1.179 ± 0.009	12.17 ± 0.13	0.50	88.06	7.44	25.86 ± 0.14	46.01 ± 0.31
3.9%	35.04 ± 0.20	2.565 ± 0.023	45.19 ± 0.48	0.23	62.48	2.00	21.93 ± 0.21	39.09±0.40
				**Plateau age** (Plateau: 3 steps from 2.0% to 2.4%)				**50.21** ± **0.15**
				**Total gas age**				48.74 ± 0.01
				**Normal isochron age** (of plateau steps)				50.25 ± 0.33
				**Inverse isochron age** (of plateau steps)				50.61 ± 0.33

^#^100% corresponds to 50 W output of CO_2_ laser. All the errors indicate 1 sigma error. ^40^Ar*: radiogenic ^40^Ar.

### Soil micromorphology

**Figure 3:**
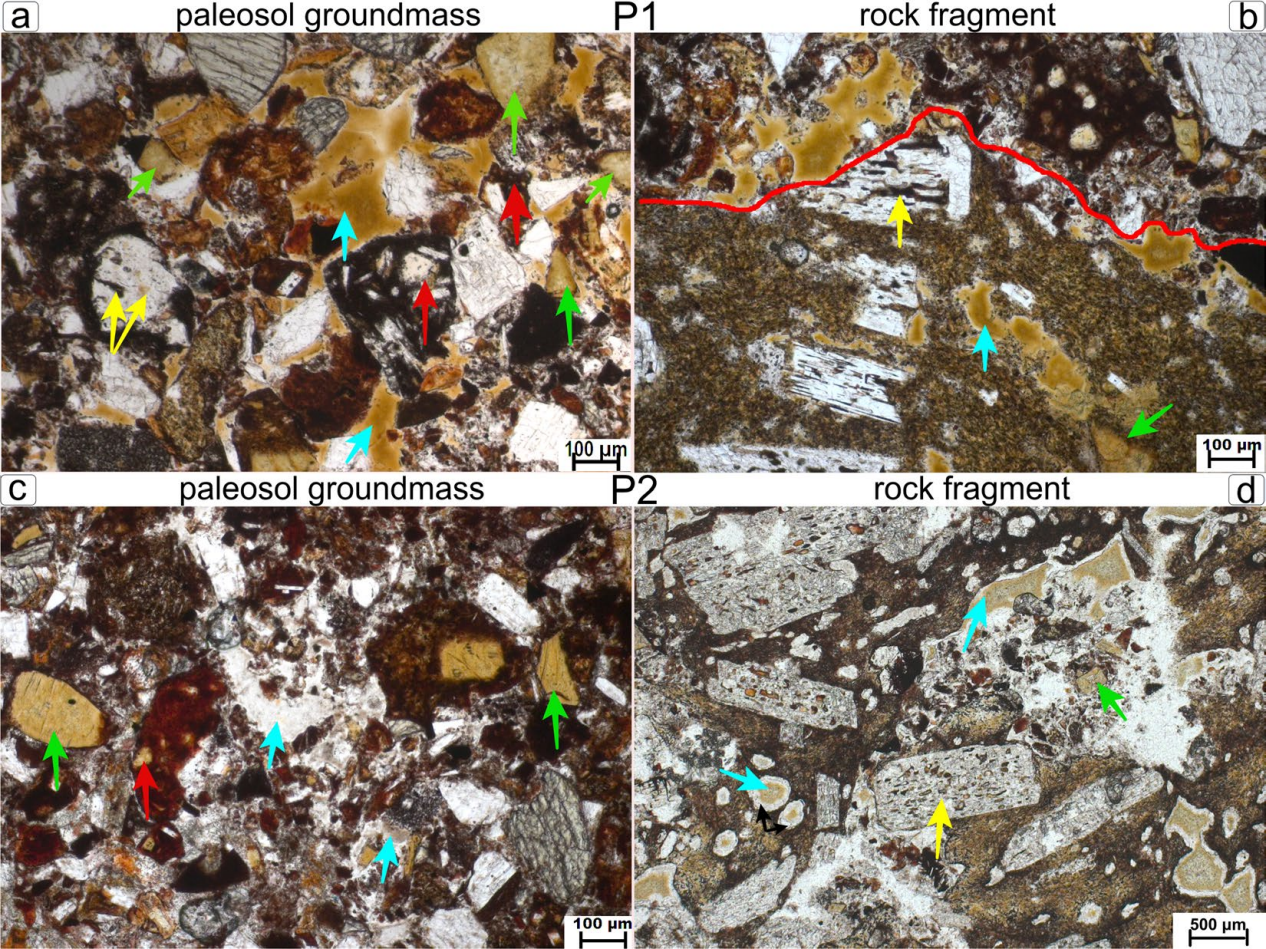
Alterations types occurring in the groundmass of paleosols and in rock fragments under plane polarized light (PPL). (**a**) P1-Bwb2, 23–34 cm: brown infillings (blue arrows); clay filling vesicles in glass shards (red arrows); brownish clays of former olivines (green arrows); yellow arrows: alterations of plagioclase (**b**) P1-Bwb2, 23–34 cm: similar alterations occurring inside a rock fragment, the red line defines the limit between rock (below the line) and paleosol (above the line). (**c**) P2-Bwb1, 20–30 cm: grey infillings are almost transparent under PPL (blue arrows); clay (red arrow) filling vesicles in glass shards; brownish clays in former olivine (green arrows). (**d**) P2-Bwb1, 20–30 cm: same alterations occurring in a rock fragment; note the brown infilling (blue arrow) surrounded by a white rim due to Fe depletion (black arrows); alteration of plagioclase (yellow arrow).

The petrographic analysis shows that the two paleosols profiles were formed on a tephra deposit rather than directly on the underlying basalt flow. The parent material is a basaltic lapilli tuff, with hypocrystalline crystallinity and porphyritic texture, consisting predominantly of plagioclases, olivines, pyroxenes and opaque phenocrysts embedded in a dark or orange groundmass (Fig. 3). Six alteration types were identified that occur both in the soil groundmass and in the rock fragments (Supplementary Table T4 and Fig. 3). Three types of alteration show a topotactic reaction or alteromorphism (transformation of a primary mineral to a secondary product with shape preservation) of olivine, plagioclase (partially transformed) and glass to clays^29^. The absence of olivine and glass in the XRD analyses indicate their complete transformation to secondary products (i.e. alteration of olivine to smectite and less frequently to serpentine). The other three clay types were neoformed and are characterized by a brown, grey and green infillings. Infillings and alteromorphs of olivine were the most frequent alterations types followed by alterations of glass and plagioclase.

Alteromorphs of primary minerals occur with similar features in both profiles. Brown clay with a mosaic-speckled b-fabric replaced olivine crystals, showing second order yellow and red interference colours under crossed polarizers (XPL). Plagioclases revealed a dotted and complex alteration pattern composed of brown, black and red clay infillings. Glass shards have mainly a cuspate to platy shape, with red, yellow, orange, brown and black colours; their voids and vesicles are often filled with brown clay. In addition, the orange and yellow shards are usually anisotropic under XPL.

Neoformation of clays occurs generally as infillings. These are a common feature in both profiles. The centre of the infilling has a speckled b-fabric (not well-oriented clay) and the boundaries of the infilling has extinction bands (well-oriented clay). These infillings are predominantly brown in P1, whereas they are mostly light-grey and nearly transparent in P2 (Fig. 3) together with brown and green colours. Another common characteristic of the infillings is the abrupt transition to the surrounding soil or rock.

Despite of the lithic discontinuities in P1, the alteration products are very similar in all horizons. The infillings are less abundant and more incorporated into the soil groundmass in the horizons 2ABb, 2BAb and 3ABb than in the upper horizons. A finer soil texture (sandy clay loam) in comparison to the horizons Bwb1, Bwb2 and Bwb3 (sandy loam), and the presence of speckled/granostriated b-fabrics in the micromass of these horizons can be taken as an evidence for a better incorporation of clays into the soil groundmass (Supplementary Table T3). Infillings are not incorporated into the soil and have well-defined borders in the horizons Bwb1, Bwb2 and Bwb3.

**Figure 4:**
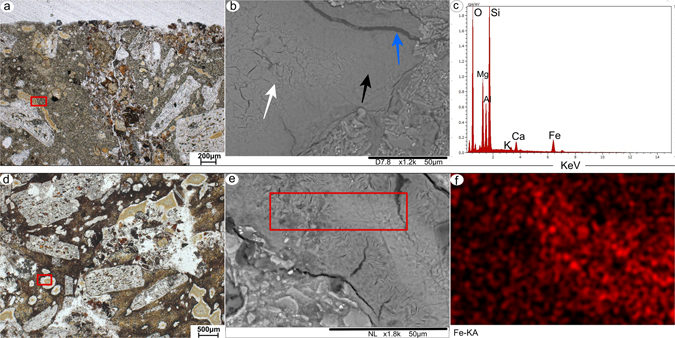
Petrographic and SEM images of selected alterations. (**a**) P1-Bwb3, 34–45 cm: brown infilling in a rock fragment (red rectangle). (**b**) the same infilling analysed with SEM. Note the abundant cracks in the centre of the infilling (white arrow) with smoother boundaries (black arrow) and a large crack next to the contact with the rock (blue arrow). (**c**) EDS spectrum of the infilling. (**d**) P2-Bwb1, 20–30 cm: brown infilling with a white rim in a rock fragment. (**e**) the same infilling analysed with SEM. Red rectangle indicate the selected area for elemental mapping (**f**) mapping of Fe in the infilling. Blackish areas indicate a lower Fe content, whereas red colours indicate a higher Fe content.

There are no major changes in the alterations types across all horizons of P2. The most relevant difference is the green colour of the clay infillings in the lowermost horizon (Bwb3), which have brown and grey colours in the other horizons. Grey infillings are predominant in the soil groundmass of the horizons Bwb1 and Bwb2, whereas infillings in the rock fragments are mostly brown, but progressively becoming grey (Figs 3 and 4). In the uppermost horizon (ABb) brown infillings turning into grey was also detected in the soil groundmass.

### SEM-EDS

The alteromorphs of olivine and the infillings (of all colours) have radial textures in wider cracks in olivines. These infillings that occur in the soil groundmass tend to be exhibit thinner cracks than those in rock fragments. Nevertheless, the overall texture of infillings in soil and rock fragments is the same. They have a centre with cracks and smoother borders and are surrounded by a large crack near the contact with the soil or rock (Fig. 4b). Such differences in texture may explain the speckled b-fabric (cracked centre), the extinction bands (smooth borders) and the abrupt interface to soil or rock (large cracks) seen under the petrographic microscope. The alterations after plagioclase and glass shards are more irregular. Primary mineral textures of plagioclase are still detectable. The transformation of glass shards into clays is better expressed around their vesicles and voids.

The EDS results show that alteromorphs of olivine having brown and green infillings are chemically similar. These types of alterations are richer in Fe and Mg, regardless if they occur in the soil groundmass or in the rock fragments (Supplementary Fig. F2). In contrast, the transitions of brown to grey infillings in P2 have a gradual loss of Fe at the boundaries of the infilling (Fig. 4f). The alteration of plagioclase reflects the composition of the primary mineral, with higher amounts of Al-Si-Ca than other alterations. The chemical composition of the major cations of volcanic shards is varying without a clear pattern.

### X-ray diffraction (XRD)

**Figure 5:**
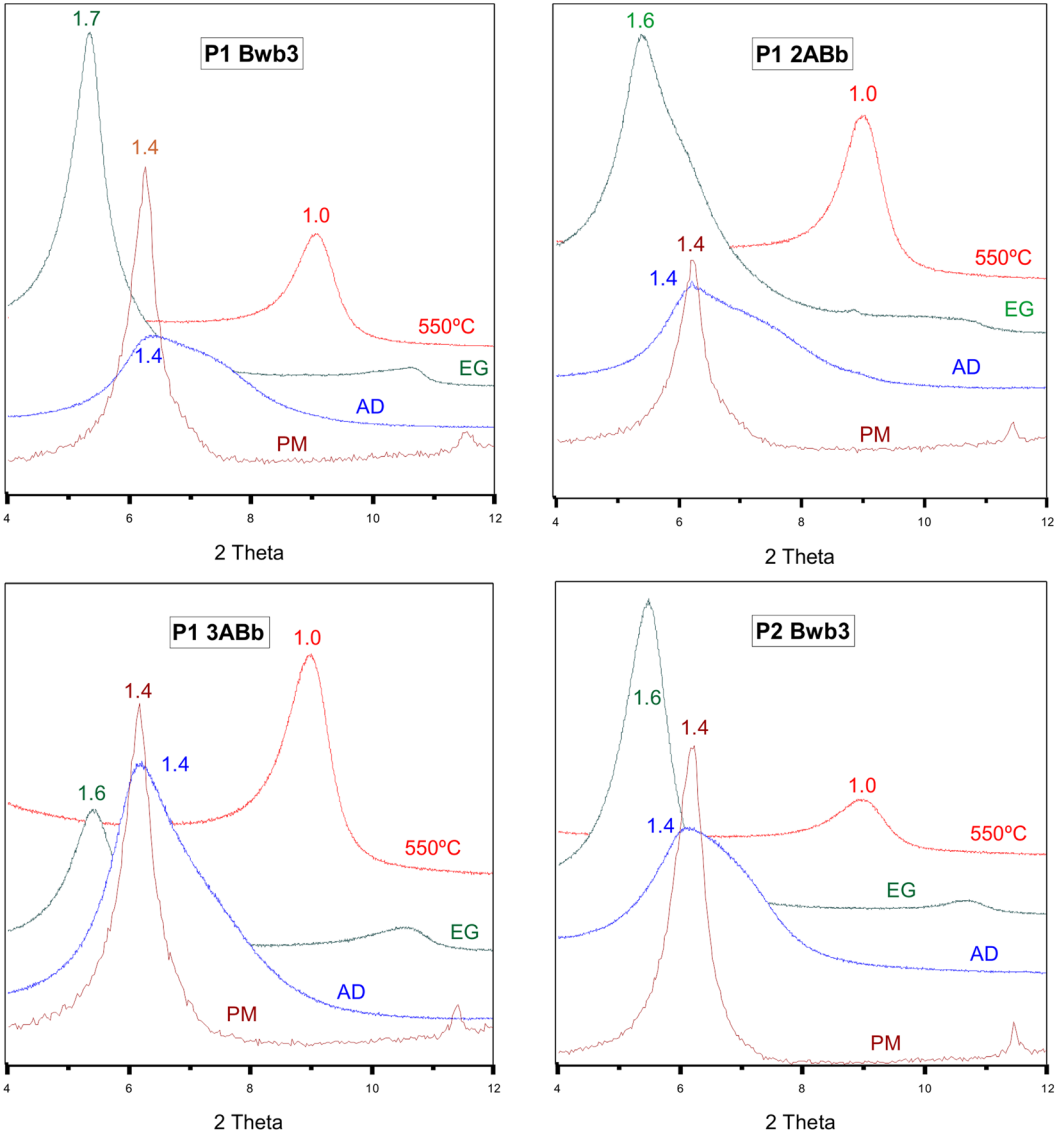
XRD patterns of clay samples from selected horizons. The d-spacing are given in nm. AD = Air dried, EG = Ethylene glycol solvation, 550 °C = sample heated at 550 °C, PM = parent material.

**Figure 6:**
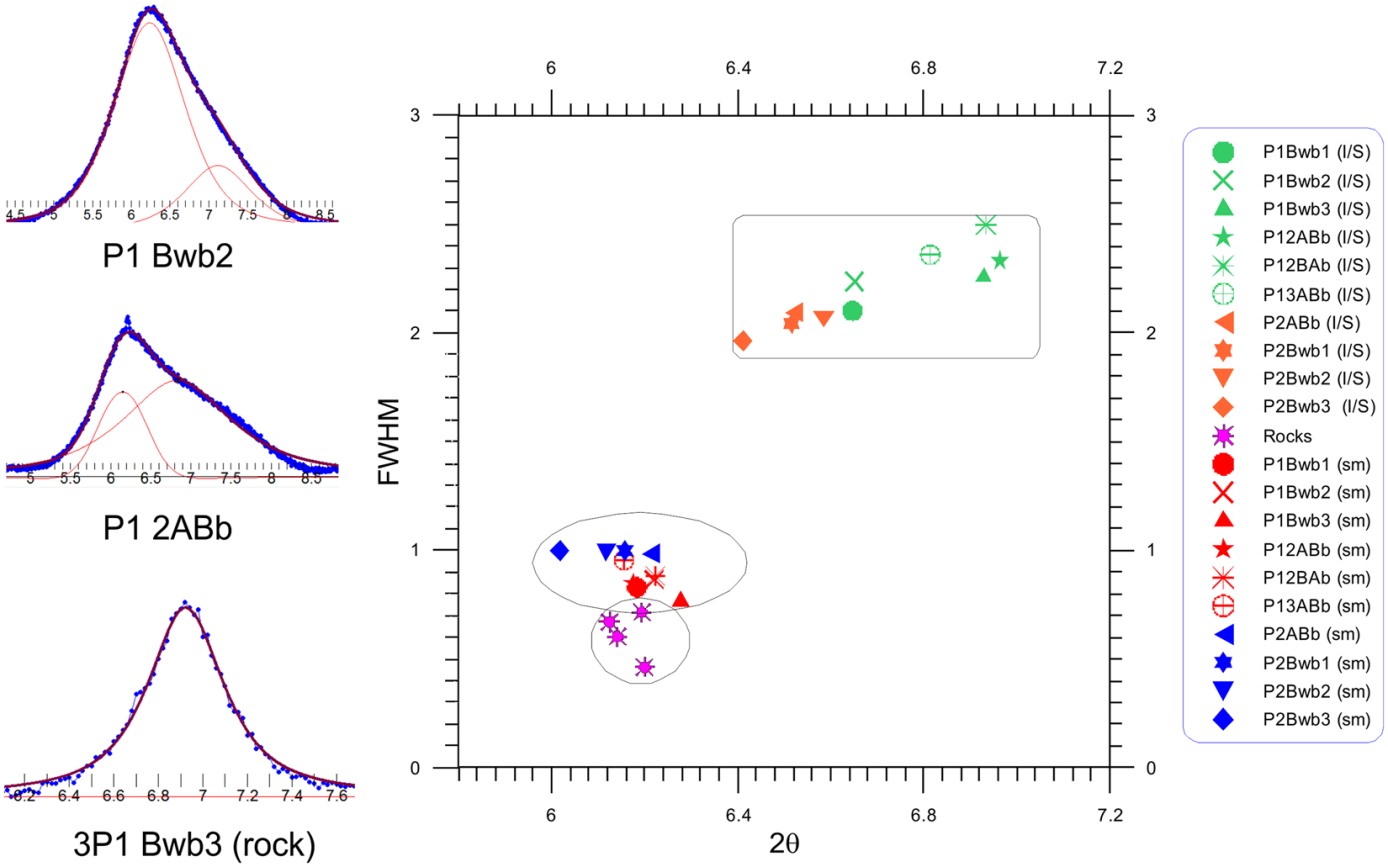
Degree of crystallinity of smectitic and interstratified components of the paleosol samples and the parent material. (**a**) Examples of profile fitting analysis obtained for clay samples of selected horizons; (**b**) Full width at half maximum (FWHM) of paleosols and rock samples. The lower the FWHM, the better the crystallinity and vice-versa. Smectitic component of P1 and P2 in red and blue, respectively. Interstratified component of P1 and P2 in green and orange, respectively. Smectite of rocks samples in pink. Each symbol represents one horizon. Note the smectitic component is more crystalline than the I/S component. Smectite from the rocks has a higher crystallinity with lower FWHM values.

The clay mineralogy of the profiles is generally homogeneous. The clays are composed of smectite having some randomly interstratified components. Smectite was confirmed by a strong *d*_001_ reflection at about 1.4 nm under air-dried conditions that shifted to about 1.7 nm after ethylene glycol treatment and collapsed to 1.0 nm after heating at 550 °C (Fig. 5). The presence of a shoulder in the *d*_001_ peak in some samples indicated a presence of randomly interstratified illite-smectite (I/S), which was better detectable after decomposing the spectra using profile fitting techniques^30^ (Fig. 6).

The main minerals in the parent material (rock fragments extracted from paleosol horizons) are plagioclase, pyroxene, quartz, and a considerable amount of smectite, reaching almost 42% in the horizon 3ABb in P1 (Table 1).

The clay association in P1 shows slight variations, having predominantly smectitic components with a minor portion of I/S. The presence of a broader shoulder of the (001) peak indicated a more developed I/S in the horizons Bwb3, 2ABb, 2BAb and 3ABb. The P2 is more homogenous and the interstratified component is only identifiable after decomposing the XRD pattern (no shoulders were visible). In both profiles, the smectitic component is better crystallized, having sharper peaks and a lower *Full Width at Half Maximum* (FWHM) value than the interstratified component. In the rock samples, the smectites have a higher degree of crystallinity, with sharper peaks (Fig. 5) and lower FWHM values than the smectitic component in the paleosols (Fig. 6).

There is a dominance of dioctahedral smectite over trioctahedral smectite with more than 80% of dioctahedral smectite in all horizons (Table 1). This is detected by the (060) reflectance on randomly oriented samples. The portions of the dioctahedral and trioctahedral smectites in the rocks samples are similar, but the dioctahedral component still predominates. An increase of the clay amount and of the proportion of dioctahedral smectite in the rock fragments can be detected in the horizons 2ABb and 3ABb of P1, which have a finer texture and less rock fragments.

Generally, there were no major differences of the clay assemblages throughout the paleosols horizons. The differences between paleosol and parent material were more perceptible. The clearest difference was a predominance of dioctahedral smectite, the presence of an interstratified component, and less crystallinity of the smectite in the paleosols.

## Discussion

While the microscopic and submicroscopic analyses showed the sources, shape and semi-quantitative chemistry of the clays, the XRD results provided the identification, degree of crystallinity and the octahedral site occupation (di- and trioctahedral) of the smectites

Alteromorphism of primary minerals, such as the plagioclases or olivine, is often used as a proof of deuteric alterations^31^. Delvigne *et al*.^32^, however, demonstrated that either deuteric alteration or weathering of olivine can produce similar products. Nonetheless, it seems that rock weathering has left the infillings and olivines alteromorphs in the soil groundmass as a residuum^33^. The alteration of plagioclase in the present study was caused by deuteric alteration. One proof is that the plagioclase phenocrysts within rock fragments are more weathered than the glassy groundmass. As a result of weathering and pedogenic processes the glassy groundmass should have been much more weathered than plagioclases in rock fragments.

The differences between the profiles are most probably related to paleodrainage conditions. The colour of the infillings in P1 is brown while in P2 is predominantly grey, with minor brown and green. In P2, the green infilling was only detected in the lowermost horizon (Bwb3), while a brown colour was present in rock fragments upwards in the profile. This suggests that the brown colour was the original colour that turned into green in Bwb3 horizon under more reducing conditions. In addition, the predominant grey colour in the soil groundmass of all horizons indicates Fe depletion, suggesting slightly reductive conditions with decreasing intensity in the uppermost horizon (ABb). In this horizon, the infillings in the soil groundmass are still brown.

Even though the horizons P1-ABb; P1-BAb and P1–3ABb have Fe nodules and have a more yellowish/greyish than reddish field colour, they generally have brown infillings. Due to weathering and biological activity these infillings are more incorporated into the soil groundmass because their boundaries are barely seen^33^. In addition, they have granostriated b-fabric^27^, which are signs of pedoturbation^34^.

The smectites in the paleosols are predominantly dioctahedral whereas the distribution with trioctahedral smectites is more balanced in the rock samples. Nevertheless, the percentage of trioctahedral smectites in the rocks is likely lower than the presented results because of the interference on the (060) peak with the quartz peak (0,15 nm) and with the basal peak of serpentine^35^. Despite this, our results are in accordance with the common knowledge that dioctahedral smectites are more common in soils because of its higher stability than the trioctahedral types^9, 36, 37^. Even in soils formed in Siberia, with limited chemical weathering, Lessovaia *et al*.^38^ reported an increase in the proportion of dioctahedral smectites in relation to trioctahedral ones from bottom to top soil horizons. Nahon *et al*.^39^ demonstrated in a study about weathering of olivine-bearing rocks that the formation of smectites started with trioctahedral species being progressively replaced by dioctahedral smectites. Some paleosols between basalts in India also had smectites formed by deuteric alteration followed by weathering^40^. Thus, it can be inferred that trioctahedral smectites in the present study were a product of deuteric alteration of the pyroclastic rocks and were progressively transformed to dioctahedral smectites during weathering in a soil environment.

The degree of crystallinity of the smectitic component is also an important difference between the paleosols and rocks samples. Pedogenic smectites tend to be less crystalline^9^, producing broader peaks or higher FWHM values, which corresponds to our findings. The smectites of the paleosols were less crystalline (higher FWHM) than those in rocks.

The presence of interstratified I/S is commonly attributed to an early stage of burial diagenesis, progressively turning smectite into illite^9, 41^. There is, however, no enrichment of K detectable in all soil horizons^27^. There are further explanations for the presence of I/S in volcanic soils/paleosols, such as inheritance from the deuteric altered parent material^11, 42^, aeolian deposition^43^ and also pedogenic, via weathering of tephra under semi-arid conditions^44^. Although all hypotheses are sound, we have no strong evidences supporting any of them.

No evidences for other typical secondary minerals of volcanic soils were found in the XRD pattern, such as nanocrystalline minerals (allophane and ferrihydrite) imogolite and halloysite. Whereas allophane is indicative of more humid climates, halloysite is often found on environments with high [Si] (silica activity), usually associated with semi-arid environments^7, 45, 46^.

The morphological similarity between smectites of the paleosols and its parent material suggests inheritance from a deuteric altered parent material as the origin. Further weathering in a soil paleoenvironment produced the differences discussed above, such as differences in crystallinity, octahedral occupancy, and interstratification. Similar clay composition between parent material and soil was previously described in other volcanic areas such as in Italy^11, 13, 47^, Costa Rica^12^ and US^10, 14^. In all these cases, the smectites were proven to be inherited from deuteric or hydrothermally altered parent material.

Homogeneity in clay assemblage and crystallinity in depth within each profile and between the profiles also support the inheritance origin^13^. A clay assemblage formed by pedogenesis should be more heterogeneous, since each soil horizon gather specific chemical-physical and biological conditions^7, 25^. Only the smectite after glass shards seems to be purely pedogenic because of heterogeneous chemistry and morphology even within the same horizon. The formation of clay inside glass vesicles also indicate authigenesis^11^. Aeolian deposition seems also unlikely or played a minor role, otherwise, a distinct signature (textural, microscopic or XRD) should be expected in the paleo A horizons.

## Conclusions

^40^Ar/^39^Ar dating was performed for the first time on these paleosols. Over- and underlying basaltic lava flows gave consistent ages ranging between 51 and 48 Ma dating the paleosols to the late Early Eocene. The mineralogical data indicate that smectites in Eocene paleosols on KGI are generally inherited from the parent material that was subjected to deuteric alterations prior to pedogenesis. The most important evidences are the homogeneity of clay assemblage between paleosols and parent material. The visual and chemical homogeneity of the clay concentrations detected under petrographic microscope and SEM/EDS and a homogeneous distribution of clays with depth in each profile supports our conclusion. Nevertheless, pedogenesis was responsible for the almost complete transformation of the inherited trioctahedral smectites (which are still present in the rocks) to dioctahedral smectites in the paleosols. Furthermore, the smectite in the paleosols are less crystalline and have an interstratified component.

The octahedral layer of the inherited smectites in the paleosols was altered by pedogenic processes, but the smectite was not further weathered to kaolinite. The imperfect drainage combined with a base-rich parent material (basaltic tephra), and likely a high pH, may have been important paleoenvironmental factors for the relative stability of the smectites. Nevertheless, since the smectite was subjected to alterations, more detailed paleoenvironmental information can potentially be taken from its isotope composition.

The question if clay minerals (e.g. smectite) are pedogenic or inherited from the parent material should be more frequently investigated in paleoenvironmental studies using clay mineralogy as a proxy since only clay minerals in soils/paleosols that are of pedogenic origin can provide paleoenvironmental information.

## Methods

### Site description and geological background

The samples were collected on King George Island, South Shetland Islands, Maritime Antarctica. The specific outcrop is located at the Cytadela area of the Ezcurra Inlet in the Admiralty Bay (62°11.057′S–58°35.209′W) (Fig. 1).

The outcrop stratigraphically belongs to the Point Thomas Formation, Ezcurra Inlet Group, which comprises a 500 m thick Paleogene (Eocene-Oligocene) volcanic succession^48, 49^. This formation was deposited during the Arctowski interglacial period (ca 50 to ca 32 Ma), a climostratigraphic unit introduced by Birkenmajer (1988)^50^.

Two informal units of the Point Thomas Formation are recognized at the Cytadela outcrop, the Lower Member (LM) and Upper Member (UM)^48^. The LM is a 20–40 m thick regular high-Al flow basalt with a thickness of 1–6 m alternating with pyroclastic deposits. The UM comprises 150–450 m of irregular, lenticular basalt lavas alternating with feldspathic tuff, interbedded with coarse vent breccia and plant−bearing tuff.

We sampled two profiles (P1 and P2), which are located in a distance of 60 m (Fig. 2). Samples were taken from paleosols below the 6^th^ basalt flow (from bottom to top) in the LM of schematic field section by Mozer (2012)^28^.

### ^40^Ar/^39^Ar dating

The groundmass samples with 250–500 µm size were prepared by crushing the fresh part of the rock, sieving, washing and the removal of phenocrysts with a magnetic separator and by handpicking. Finally, the samples are soaked in 1 N HCl for a few minutes to remove secondary minerals on their surface.

Neutron activation of the groundmass was performed at the Oregon State TRIGA Reactor (OSTR) in the University of Oregon, USA. The samples were irradiated by the fast neutrons in the CLICIT facility, in which a Cd tube with 0.51 mm thickness is equipped. Samples were wrapped by commercial Al foils, then were loaded into the 99.999% pure Al sample container together with the neutron-flux monitoring mineral, Fish Canyon Tuff sanidine (FC3), prepared by Geological Survey of Japan (27.5 Ma^51, 52^); crystals of K_2_SO_4_ and CaF_2_ for correction of interference by the Ar isotopes produced from K and Ca in the samples. They all had been irradiated for 4 hours with the fast neutron flux of 2.5 × 10^13^ n/cm^2^/s. After cooling of samples, they were sent back to Potsdam then their Ar isotopes were analysed at the ^40^Ar/^39^Ar geochronology laboratory in the University of Potsdam^53, 54^.

The Ar isotopic analytical system consists of, (1) a New Wave Gantry Dual Wave laser ablation system with a 50 W CO_2_ laser (wavelength 10.6 micrometre) for heating samples and extracting gases, (2) an ultra-high vacuum purification line equipped with SAES getters and the cold trap held at the frozen temperature of ethanol, and (3) a high-sensitivity Micromass 5400 noble gas mass spectrometer equipped with an electron multiplier conducting the pulse-counting analysis. All groundmass samples of about 13 mg each were firstly loaded into the sample chamber. Then the sample chamber and the purification line were baked for 24 hours at 100 and 200 °C. The groundmass samples were firstly preheated and pumped with 1.2% output (0.6 W), then were analysed by stepwise heating for two minutes with 10–15 steps with a continuous CO_2_ laser beam with 1.5 mm diameter. The obtained Ar isotope ratios by the analysis are corrected for blank, mass discrimination, interferences and decay corrections following Uto *et al*.^51^, and then the plateau age, total gas age, normal and inverse isochron ages are calculated for each sample. Decay constants and atmospheric Ar isotope ratios follow Steiger and Jäger (1977)^55^ and the calculation of isochron ages follows York (1969)^56^. Plateau ages were determined by the criteria of Fleck *et al*.^57^. Finally concluded ages for each sample were obtained by the comparison among plateau age, normal and inverse isochron ages from plateau steps or all steps and total gas age with considering if the initial ^40^Ar/^36^Ar ratios obtained by isochrons were valid. All results not shown in the text (i.e. Fig. 2 and Table 2) are in Supplementary Fig. F1 and Supplementary Table T2.

### Micromorphological and SEM-EDS analyses

We described 10 undisturbed and oriented samples. Detailed micromorphological description can be found in the Supplementary Table T3. The analysis were made in 9 × 6 cm thin sections and photographed using a polarizing microscope (Zeiss Axio Imager.A2m, Software AxioVision 4.7.2) with plane polarized light (PPL) and crossed polarized light (XPL). The most important alteration types were grouped into six categories (Table 2). The textures and microchemistry of the alterations were determined using a Hitachi TM3030 Plus Scanning electron microscope (SEM) coupled with a Bruker Quantax 70 X-ray microanalysis detector (EDS) on polished uncoated thin sections at the Institute of Mineralogy and Geodynamics, University of Tübingen.

### X-ray diffraction (XRD)

We analyzed the clay fraction (<2 μm) of all horizons and the coarse fraction (>2 mm) of selected horizons in a total of 10 and 4 samples, respectively. The coarse fraction is the rocks fragments (pyroclastic material) forming the paleosol parent material.

The clay fraction was separated in distilled water according to Stoke’s law and was prepared using two different methods:

- air-dried oriented preparations were obtained by pipetting some drops of the suspensions onto a glass slide, which was then dried at 30 °C for a few hours^35^. Ethylene glycol solvation of the slides was achieved by exposing them to ethylene glycol vapor at 70 °C for a minimum of 12 hours.
- randomly oriented samples, to measure the 060 reflections that allowed the distinction between dioctahedral and trioctahedral clay minerals, were prepared using a back-loading procedure^35^.

Measurements were made using an Empyrean diffractometer operating with an accelerating voltage of 45 V and a filament current of 30 mA, using CuK_α_ radiation, nickel filter and PixCELL 3D detector.

Oriented samples were examined by XRD in the air-dried form, saturated with ethylene glycol (EG) and after heating (550 °C). The preparations were measured over a 2θ angle range of 2–70° (air-dried) and 2–30° (glycolated and heated) with a step size of 0.04° (2θ) and 40 s of scan step time. For randomly samples the peaks were resolved from the background by small step scan (0.002° 2θ) and long count time (100 s) measurements.

Profile fitting was calculated from oriented samples using simple peak weighting factors. For area estimation, we used *Fityk* ^30^, a software for data processing and nonlinear curve fitting, simple background subtraction and easy placement of peaks and changing of peak parameters. The smectite peak was evaluated by peak fitting that is based on a pseudo-Voigt function.

The decomposition of peaks using profile fitting^30, 58^ allows the identification of clay assemblage and provides information of peak position and full width at half maximum intensity (FWHM). For the FWHM measurement, instrumental broadening effects were calibrated via a LaB6 standard (NIST 660a).

## Electronic supplementary material

Supplementary information
