# Supplementary material for: Origin of clay minerals in Early Eocene volcanic paleosols on King George Island, Maritime Antarctica
Source: Sci Rep. 2017 Jul 25;7:6368. doi: 10.1038/s41598-017-06617-x (PMC5526868; doi:10.1038/s41598-017-06617-x)
Supplement: Supplementary file 1 — Supplementary information [file 41598_2017_6617_MOESM1_ESM.pdf]

Origin of clay minerals in Early Eocene volcanic paleosols on King George Island, Maritime Antarctica

Diogo Noses Spinola, Teresa Pi-Puig, Elizabeth Solleiro-Rebolledo, Markus Egli, Masafumi Sudo, Sergey Sedov, Peter Kühn

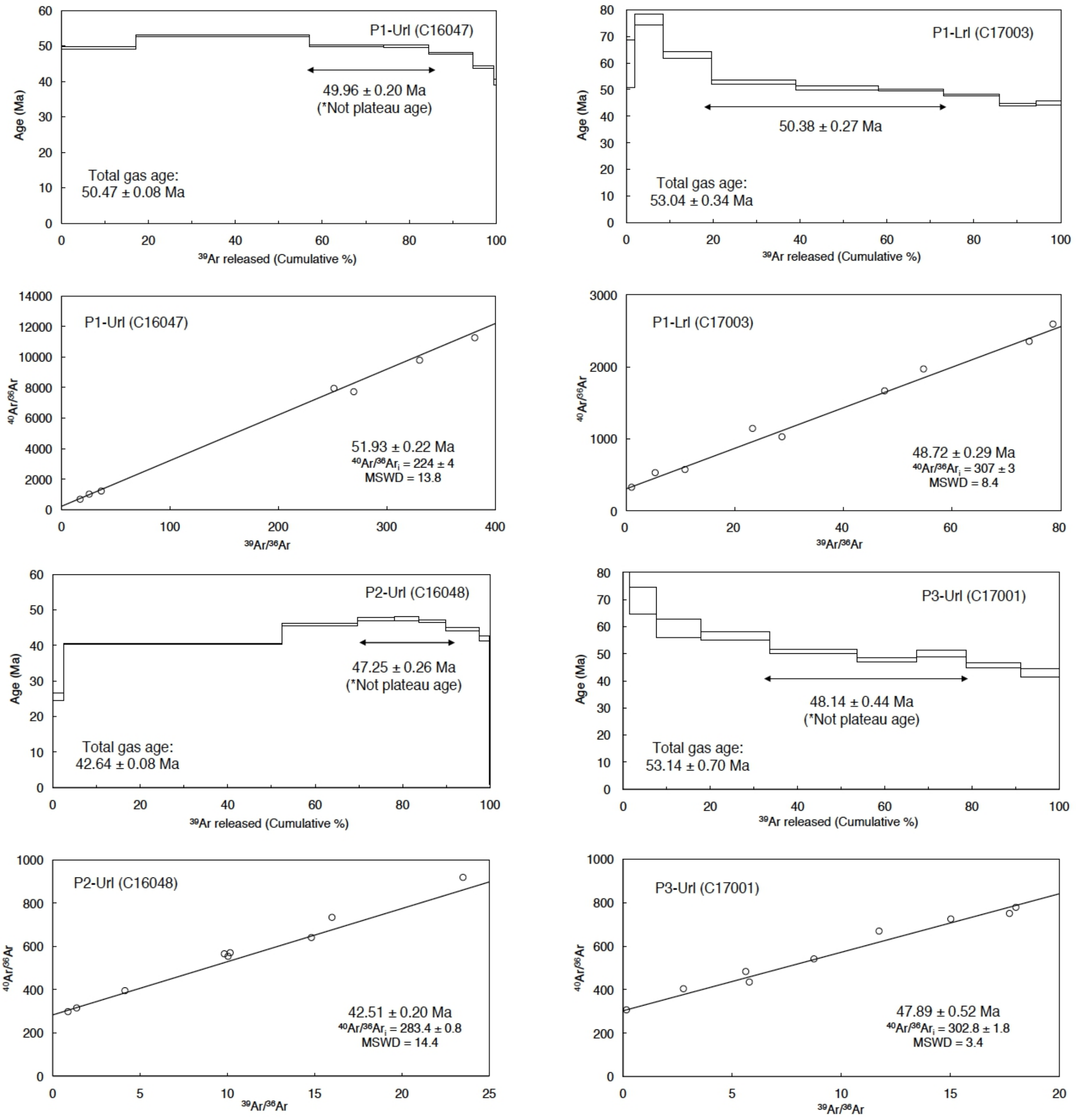

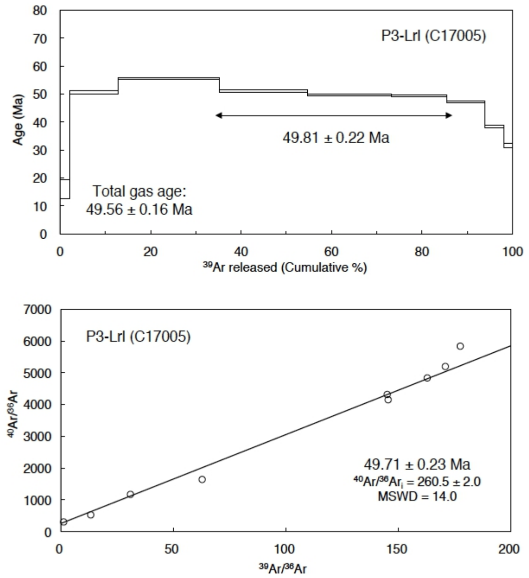

Supplementary Figure F1. $^{40}Ar/^{39}Ar$ age spectra and normal isochron plots obtained by the stepwise heating analysis of the other five groundmass samples than the sample P2-Lrl shown in Fig. 2 in the text. Sample-ID and Laboraotry-ID are indicated in all figures. Closed circles in the isochron plot are derived from plateau steps instead open circles are not.

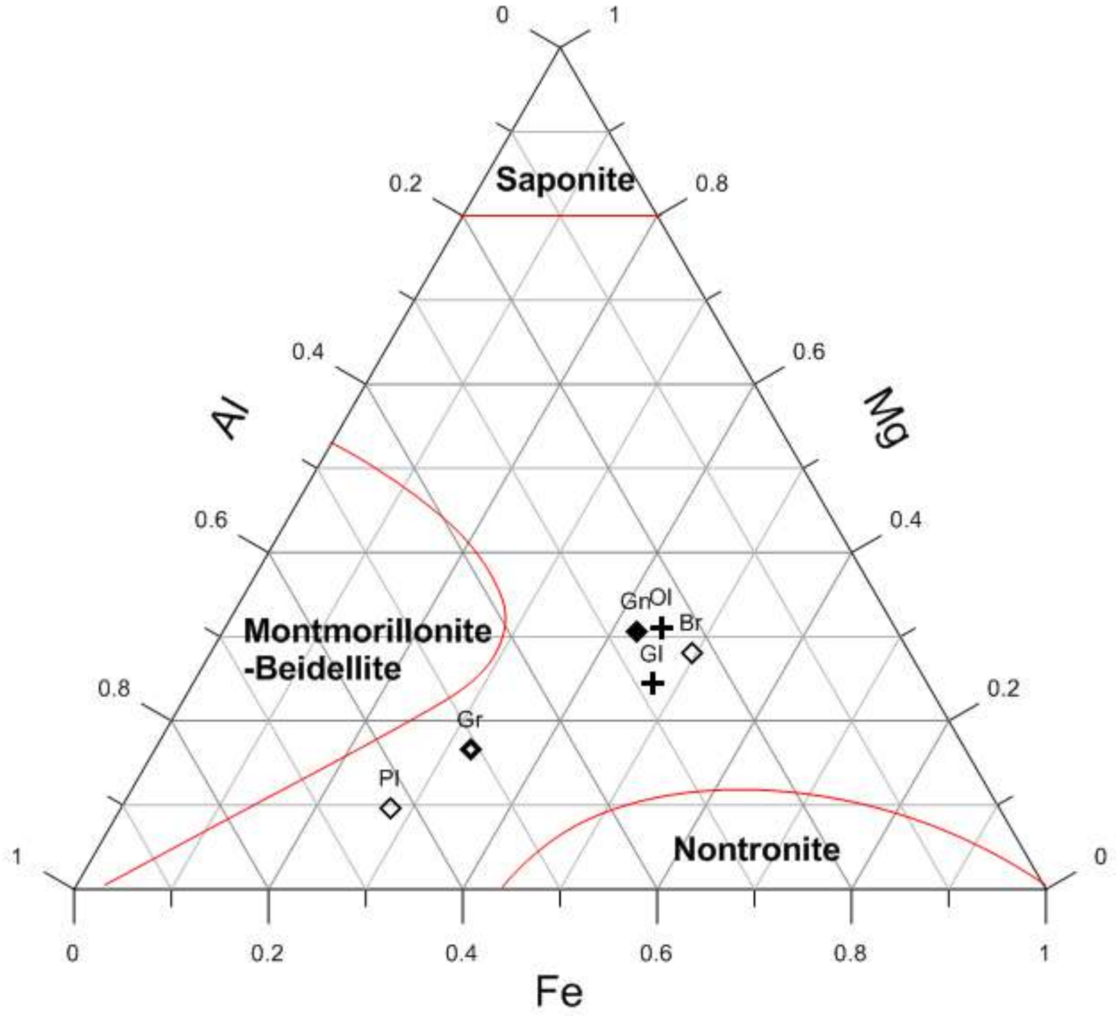

Supplementary Figure F2. Fe_2O_3 – Al_2O_3 – MgO Ternary diagram with the average chemical composition (EDS values) of each alteration. Br = brown infilling; Gr = grey infilling; Gn = green infilling; Ol = olivine alteration; Pl = plagioclase alteration; Gl = glass alteration.

Supplementary Table T1. Selected main macromorphological properties (after FAO, 2006).

Profile	Horizon	Depth	Horizon boundaries (distinctness - topography	Structure[1]		Colour (Munsell)		Rock fragments	Plant fossil	Particle size %			Textural class
		[cm]		Type	Size	Dry	Name			Clay (<2µ)	Silt (2 µ-63 µ)	Sand (63µ - 2000µ	
**P1**	Bwb1	0 - 23	clear - wavy	sb +pl	very fine	5YR 5/4	Dull reddish brown	many		15	18	67	Sandy loam
	Bwb2	23 - 34	gradual – smooth	sb+pl	fine/thin	2.5YR 5/4	Dull reddish brown	common		15	19	66	Sandy loam
	Bwb3	34 - 45	abrupt- smooth	ab	fine	2.5YR 5/4	Dull reddish brown	common		14	19	67	Sandy loam
	2ABb	45 - 60	clear - smooth	ab	fine	7.5YR 7/4	Dull orange	none		17	21	62	Sandy loam
	2BAb	60 - 73	abrupt - smooth	ab+pl	coarse	10YR 6/4	Dull yellow orange	none		20	22	58	Sandy clay loam
	3ABb	73+		pl+ab	medium	5Y 4/1	Gray	none	x	20	15	65	Sandy clay loam
**P2**	ABb	0 - 20	abrupt - smooth	sb	medium	2.5YR 5/4	Dull reddish brown	few		19	17	64	Sandy clay loam
	Bwb1	20 - 30	gradual - smooth	ab+pl	coarse	2.5YR 2/4	Reddish	few		15	19	66	Sandy loam
	Bwb2	30 - 53	diffuse - irregular	ab+pl	medium	2.5YR 5/4	Dull reddish brown	common		15	18	67	Sandy loam
	Bwb3	53+		ab+sb	medium	2.5YR 5/4	Dull reddish brown	many		15	13	72	Sandy loam

[1]**Structure type:** ab= angular blocky., sb= subangular blocky., pl= platy. **Size**: vf= very fine/thin., fi= fine/thin., me=medium., co= coarse/thick

Supplementary Table T2. $^{40}Ar/^{39}Ar$ analytical results of basaltic lava flows, P1-Url, P1-Lrl, P2-Url, P3-Url and P3-Lrl.

Sample ID: P1-Url *Laboratory ID: C16047* *Neutron irradiation ID: PO-5*

J= #######

Laser output	$^{40}Ar/^{39}Ar$			$^{37}Ar/^{39}Ar$			$^{36}Ar/^{39}Ar$ (x10^{-3})			K/Ca	$^{40}Ar^*$ (%)	$^{39}Ar_K$ Fraction (%)	$^{40}Ar^*/^{39}Ar_K$			Age (±1 σ) (Ma)		
1.6%	40.00	±	0.19	0.361	±	0.003	39.24	±	0.28	1.63	71.08	17.10	28.44	±	0.16	49.44	±	0.34
2.0%	31.59	±	0.04	0.521	±	0.001	4.12	±	0.02	1.13	96.28	39.85	30.43	±	0.04	52.85	±	0.22
2.2%	29.51	±	0.09	0.942	±	0.005	2.88	±	0.04	0.62	97.37	17.12	28.75	±	0.09	49.98	±	0.25
2.4%	29.59	±	0.14	1.265	±	0.007	3.37	±	0.07	0.46	96.98	10.46	28.72	±	0.14	49.93	±	0.31
2.8%	28.61	±	0.16	1.640	±	0.010	4.15	±	0.08	0.36	96.17	10.11	27.55	±	0.16	47.91	±	0.34
3.4%	33.29	±	0.23	2.451	±	0.017	27.90	±	0.29	0.24	75.83	4.74	25.28	±	0.20	44.02	±	0.39
4.0%	39.86	±	0.29	5.778	±	0.053	59.70	±	1.42	0.10	56.91	0.62	22.77	±	0.47	39.69	±	0.82

**Plateau age**	(No Plateau)		±	
**Total gas age**		50.47	±	0.08
**Normal isochron age**	(of all steps)	51.93	±	0.22
**Inverse isochron age**	(of all steps)	52.52	±	0.22

Sample ID: P1-Lrl *Laboratory ID: C17003* *Neutron irradiation ID: PO-5*

J= #######

Laser output	$^{40}Ar/^{39}Ar$			$^{37}Ar/^{39}Ar$			$^{36}Ar/^{39}Ar$ (x10^{-3})			K/Ca	$^{40}Ar^*$ (%)	$^{39}Ar_K$ Fraction (%)	$^{40}Ar^*/^{39}Ar_K$			Age (±1 σ) (Ma)		
1.4%	345.24	±	6.14	1.613	±	0.049	######	±	####	0.36	10.03	1.93	34.66	±	5.27	59.83	±	8.96
1.6%	100.1	±	1.5	1.86	±	0.03	189	±	2	0.32	44.33	6.55	44.4	±	1.2	76	±	2
1.8%	49.2	±	0.8	2.42	±	0.04	43.6	±	0.7	0.24	74.18	11.07	36.6	±	0.7	63.1	±	1.2
2.1%	35.9	±	0.5	3.83	±	0.05	19.3	±	0.3	0.15	85.00	19.39	30.6	±	0.4	52.9	±	0.8
2.4%	32.9	±	0.5	5.33	±	0.08	14.1	±	0.2	0.11	88.60	18.98	29.3	±	0.4	50.6	±	0.8
2.7%	34.91	±	0.15	6.06	±	0.04	22.60	±	0.18	0.10	82.27	15.13	28.83	±	0.14	49.9	±	0.3
3.1%	31.6	±	0.2	7.06	±	0.05	15.35	±	0.18	0.08	87.44	12.76	27.73	±	0.19	48.0	±	0.4
3.6%	35.7	±	0.3	10.46	±	0.10	37.5	±	0.5	0.06	71.29	8.45	25.6	±	0.3	44.4	±	0.5
4.2%	52.8	±	0.5	13.80	±	0.14	95.7	±	0.9	0.04	48.61	5.74	25.9	±	0.4	44.9	±	0.7

**Plateau age**	(Plateau: 3 steps from2.1% to 2.7%)	50.38	±	0.27
**Total gas age**		53.04	±	0.34
**Normal isochron age**	(of all steps)	48.72	±	0.29
**Inverse isochron age**	(of all steps)	48.57	±	0.29

Sample ID: P2-Url *Laboratory ID: C16048* *Neutron irradiation ID: PO-5*

J= #######

Laser output	$^{40}Ar/^{39}Ar$			$^{37}Ar/^{39}Ar$			$^{36}Ar/^{39}Ar$ (x10^{-3})			K/Ca	$^{40}Ar^*$ (%)	$^{39}Ar_K$ Fraction (%)	$^{40}Ar^*/^{39}Ar_K$			Age (±1 σ) (Ma)		
1.4%	230.86	±	1.16	0.370	±	0.006	731.7	±	3.8	1.59	6.35	2.51	14.67	±	0.60	25.46	±	1.04
1.6%	43.30	±	0.04	0.291	±	0.001	67.6	±	0.1	2.02	53.93	49.97	23.36	±	0.03	40.38	±	0.17
1.8%	39.15	±	0.15	0.275	±	0.002	42.6	±	0.2	2.14	67.87	17.17	26.57	±	0.13	45.87	±	0.28
2.0%	45.94	±	0.24	0.462	±	0.003	62.7	±	0.3	1.27	59.78	8.46	27.47	±	0.20	47.40	±	0.39
2.3%	57.61	±	0.32	0.601	±	0.004	101.9	±	0.6	0.98	47.80	5.61	27.55	±	0.28	47.52	±	0.52
2.7%	56.19	±	0.30	0.923	±	0.008	98.7	±	0.5	0.64	48.25	6.14	27.13	±	0.25	46.81	±	0.46
3.2%	55.19	±	0.28	1.877	±	0.012	100.0	±	0.6	0.31	46.73	7.67	25.82	±	0.25	44.58	±	0.47
3.7%	95.53	±	0.49	3.607	±	0.022	242.4	±	1.3	0.16	25.32	2.30	24.24	±	0.42	41.89	±	0.73
4.2%	343.02	±	4.26	4.044	±	0.067	1150.1	±	15.4	0.15	1.02	0.17	3.49	±	2.95	6.10	±	5.13

**Plateau age**	(No Plateau)		±	
**Total gas age**		42.64	±	0.08
**Normal isochron age**	(of all steps)	42.51	±	0.20
**Inverse isochron age**	(of all steps)	42.66	±	0.20

Sample ID: P3-Url *Laboratory ID: C17001* *Neutron irradiation ID: PO-5*

J= #######

Laser output	$^{40}Ar/^{39}Ar$			$^{37}Ar/^{39}Ar$			$^{36}Ar/^{39}Ar$ (x10^{-3})			K/Ca	$^{40}Ar^*$ (%)	$^{39}Ar_K$ Fraction (%)	$^{40}Ar^*/^{39}Ar_K$			Age (±1 σ) (Ma)		
1.4%	2006.4	±	19.4	8.7	±	0.2	6539.6	±	66.5	0.07	3.72	1.49	75.05	±	12.38	130.55	±	20.79
1.6%	145.7	±	2.8	5.6	±	0.1	362.1	±	8.8	0.10	26.88	6.13	39.31	±	2.91	69.57	±	5.06
1.8%	85.5	±	2.1	8.3	±	0.2	179.2	±	5.4	0.07	38.86	10.16	33.43	±	1.99	59.32	±	3.48
2.0%	56.6	±	1.0	11.0	±	0.2	87.6	±	2.0	0.05	55.85	15.87	31.86	±	0.90	56.59	±	1.60
2.2%	47.8	±	0.5	13.9	±	0.2	69.8	±	0.8	0.04	59.23	19.91	28.58	±	0.43	50.84	±	0.78
2.4%	42.8	±	0.5	16.2	±	0.2	59.4	±	0.9	0.04	62.05	13.80	26.83	±	0.45	47.77	±	0.82
2.7%	61.2	±	0.8	16.4	±	0.2	117.5	±	1.9	0.04	45.41	11.30	28.10	±	0.73	50.00	±	1.29
3.2%	41.8	±	0.6	21.1	±	0.3	61.4	±	1.0	0.03	60.62	12.61	25.69	±	0.54	45.76	±	0.97
3.8%	73.6	±	1.0	31.8	±	0.4	177.9	±	2.5	0.02	32.05	8.72	24.09	±	0.82	42.94	±	1.45

**Plateau age**	(No Plateau)		±	
**Total gas age**		53.14	±	0.70
**Normal isochron age**	(of all steps)	47.89	±	0.52
**Inverse isochron age**	(of all steps)	48.21	±	0.52

Sample ID: P3-Lrl *Laboratory ID: C17005* *Neutron irradiation ID: PO-5*

J= #######

Laser output	$^{40}Ar/^{39}Ar$			$^{37}Ar/^{39}Ar$			$^{36}Ar/^{39}Ar$ (x10^{-3})			K/Ca	$^{40}Ar^*$ (%)	$^{39}Ar_K$ Fraction (%)	$^{40}Ar^*/^{39}Ar_K$			Age (±1 σ) (Ma)		
1.4%	267.44	±	2.67	1.52	±	0.04	875.27	±	7.86	0.39	3.34	2.06	8.93	±	1.86	16.04	±	3.32
1.6%	38.01	±	0.47	0.87	±	0.01	32.63	±	0.38	0.67	74.81	10.69	28.45	±	0.42	50.62	±	0.76
1.8%	32.85	±	0.15	1.04	±	0.01	5.91	±	0.07	0.57	94.94	22.36	31.21	±	0.15	55.44	±	0.34
2.0%	30.36	±	0.25	2.26	±	0.02	6.46	±	0.09	0.26	94.31	19.52	28.68	±	0.24	51.01	±	0.46
2.2%	29.64	±	0.19	3.32	±	0.02	7.04	±	0.09	0.18	93.89	18.69	27.89	±	0.18	49.63	±	0.38
2.4%	29.68	±	0.16	4.20	±	0.02	8.02	±	0.12	0.14	93.15	12.21	27.72	±	0.15	49.33	±	0.33
2.7%	28.43	±	0.15	5.10	±	0.03	8.25	±	0.15	0.11	92.88	8.27	26.50	±	0.15	47.18	±	0.32
3.1%	26.04	±	0.28	8.37	±	0.09	18.14	±	0.31	0.07	82.01	4.33	21.48	±	0.27	38.34	±	0.50
3.7%	39.58	±	0.48	17.45	±	0.25	79.59	±	1.26	0.03	44.13	1.88	17.67	±	0.48	31.60	±	0.87

**Plateau age**	(Plateau: 3 steps from2.0% to 2.4%)	49.81	±	0.22
**Total gas age**		49.56	±	0.16
**Normal isochron age**	(of all steps)	49.71	±	0.23
**Inverse isochron age**	(of all steps)	50.83	±	0.24

[#] 100% corresponds to 50W output of CO_2 laser. All the errors indicate 1 sigma error. $^{40}Ar^*$: radiogenic ^{40}Ar

Supplementary Table T3. Selected main micromorphological characteristics.

**Profile**	**Horizon**	**Depth**	**Pedality**			**Voids**							**Accomodation**			**Microstructure**	**Micromass**					**Plant residue**	**Pedofeatures**			**Zeolite**
																	Colour		b-fabric							
		[cm]	sp	mp	wp	spv	cmv	cxp	ch	cm	ve	vu	a	pa	un		ppl	oil	u	ssp	gs		cc	pf	Fe/Mn nod	
**P1**	Bwb1	0 - 23	x			x			x	x		x	x	x	x	sb, (gr-cr)	br, rd, lb, bl	rd	x				x	x		x
	Bwb2	23 - 34			x						x		x			(sb)	br, rd, lb, bl	rd	x					x		
	Bwb3	34 - 45		x					x				x	x	x	sb, (gr)	br, rd, lb, bl	rd	x					x		
	2ABb	45 - 60			x				x				x			(sb)	lb, br, rd	lb, br	x	(x)	(x)	x		x	x	
	2BAb	60 - 73		x								x	x			(gr)	lb, br, rd	lb, br	x	(x)	(x)	x		x	x	
	3ABb	73+															gr, lb, br	ol, lb	x	(x)	(x)			x	x	
**P2**	ABb	0 - 20		x		x	x	x	x			x	x	x	x	sb, gr	br, lb, rd, bl	rd	x			x			x	x
	Bwb1	20 - 30		x					x			x	x	x	x	sb, (gr-cr)	br, lb, rd, bl	rd	x					x	x	
	Bwb2	30 - 53			x				x		x	x	x			(sb)	br, lb, rd, bl	rd	x					x	x	
	Bwb3	53+			x				x			x	x	x		(sb)	br, lb, rd, bl	rd	x					x	x	

The micromorphological property is shown by the presence (cross) or absence (no cross).
The brackets are used when the property is weakly expressed

Pedality: sp=strongly developed pedality., mp=moderately developed., wp=weakly developed

Voids: spv= simple packing voids., cmv= compound packing voids., cxp= complex packing voids., ch= channels., cm= chamber., ve= vesicles., vu= vughs

Accomodation of planar voids: a= accommodated., pa= partially accommodated., un= unaccommodated

Microstructure: sb= subangular., gr= granular., cr= crumb

Colour: PPL (Plane Polarized Light); OIL (Oblique Incident Light): rd= red., br= brown., bl= black., lb= light brown., ol= olive, gr= grey

b-fabric: u= undifferentiated., ssp= stipple speckled., gs= granostriated

Pedofeatures: cc = clay coatings, pf= passage features, Fe/Mn nod = iron/manganese nodules

Supplementary Table T4. Alteration types distribution through the paleosols horizons.

Profile	Horizon	Depth (cm)	Alteration type					
			Transformation*			Neoformation**		
			Olivine	Plagioclase	Glass	Brown infiling	Grey infilling	Green infilling
P1	Bwb1	0-23	x	x	x	x		
	Bwb2	23-34	x	x	x	x		
	Bwb3	34-45	x	x	x	x		
	2ABb	45-60	x	x	x	x		
	2BAb	60-73	x	x	x	x		
	3ABb	73+	x	x	x	x		
P2	ABb	0-20	x	x	x	x	x	
	Bwb1	20-30	x	x	x	(x)	x	
	Bwb2	30-53	x	x	x	(x)	x	
	Bwb3	53+	x	x	x	(x)	x	x
